# Density of Common Complex Ocular Traits in the Aging Eye: Analysis of Secondary Traits in Genome-Wide Association Studies

**DOI:** 10.1371/journal.pone.0002510

**Published:** 2008-06-25

**Authors:** Albert O. Edwards, Sung J. Lee, Brooke L. Fridley, Nirubol Tosakulwong

**Affiliations:** 1 Department of Ophthalmology, Mayo Clinic, Rochester, Minnesota, United States of America; 2 Division of Biostatistics, Mayo Clinic, Rochester, Minnesota, United States of America; Vrije Universiteit Medical Centre, Netherlands

## Abstract

Genetic association studies are identifying genetic risks for common complex ocular traits such as age-related macular degeneration (AMD). The subjects used for discovery of these loci have been largely from clinic-based, case-control studies. Typically, only the primary phenotype (e.g., AMD) being studied is systematically documented and other complex traits (e.g., affecting the eye) are largely ignored. The purpose of this study was to characterize these other or secondary complex ocular traits present in the cases and controls of clinic-based studies being used for genetic study of AMD. The records of 100 consecutive new patients (of any diagnosis) age 60 or older for which all traits affecting the eye had been recorded systematically were reviewed. The average patient had 3.5 distinct diagnoses. A subset of 10 complex traits was selected for further study because they were common and could be reliably diagnosed. The density of these 10 complex ocular traits increased by 0.017 log-traits/year (P = 0.03), ranging from a predicted 2.74 at age 60 to 4.45 at age 90. Trait-trait association was observed only between AMD and primary vitreomacular traction (P = 0.0009). Only 1% of subjects age 60 or older had no common complex traits affecting the eye. Extrapolations suggested that a study of 2000 similar subjects would have sufficient power to detect genetic association with an odds ratio of 2.0 or less for 4 of these 10 traits. In conclusion, the high prevalence of complex traits affecting the aging eye and the inherent biases in referral patterns leads to the potential for confounding by undocumented secondary traits within case-control studies. In addition to the primary trait, other common ocular phenotypes should be systematically documented in genetic association studies so that adjustments for potential trait-trait associations and other bias can be made and genetic risk variants identified in secondary analyses.

## Introduction

The eye has long been an excellent organ for studying hereditary diseases, in part, because the visual system enables use of our discriminating sense of vision to characterize phenotypes. Genetic association studies on age-related macular degeneration (AMD, Online Mendelian Inheritance of Man, OMIM, 603075), cataract (OMIM 601371), glaucoma (OMIM 137760), and other diseases are unraveling the genetic risks for common complex ocular traits [1]–[3]. Other common diseases such as epiretinal membranes are not known to be hereditary, but could influence study endpoints through biases in referral patterns or unknown genetic effects. Genetic association studies have proven a powerful method and important genetic risks have been identified for systemic age-related complex traits such as cancer and coronary artery disease [4], [5]. Many of these studies use clinic-based case-control subjects ascertained for the presence or absence of the primary trait (e.g., AMD) and systematically document phenotypes on few, if any, other complex ocular traits [6]–[10].

The unavailability of phenotypes on other (secondary) ocular traits in a case-control study gives rise to a number of limitations. Because such clinic based studies are collected from a number of different subspecialty clinics, the inability to account for the diagnoses for which the patient presented could lead to undetected confounding of genetic results. Trait-trait associations that might confound the interpretation of association results, such as the reported increased risk of exudative AMD in eyes with posterior vitreous adherence to the macula or Fuchs endothelial dystrophy (OMIM 610158), cannot be assessed [11]–[14]. Genetic risks may alter the risk of related diseases, such as the common risk on chromosome 8q24 for cancer, and it is well established that Mendelian diseases can affect multiple tissues with diseases indistinguishable from the complex traits affecting the eye [4], [15], [16]. Such shared risks are likely to be missed in the absence of systematic phenotyping of common traits affecting an organ.

Equally important to these potential problems is the inability to explore genotypes generated in genome-wide studies for association with secondary ocular traits or even the large number of biomorphic and biochemical variables such as axial length of the eye, corneal thickness, and retinal autofluorescence that influence disease progression [17]–[19]. Indeed, some common complex traits, biomorphic features, and biochemical endpoints might never be the subject of a dedicated study because of a perceived lack of value due to the high cost of genetic association studies and the low rate of uncorrectable vision loss due to mild severity or the availability of surgical interventions.

A great deal of work goes into the ascertainment of subjects for disease-specific case-control studies. Some of this effort is specific to each disease. However other factors such as fundus photographs of the macula in studies of AMD can also be used to study the retinal vasculature (e.g., occlusions) and optic nerve (e.g., glaucoma). Similarly, activities such as consent, eye examination, phlebotomy, isolation of genomic DNA, and genotyping would apply to all traits initially. Environmental exposures such as tobacco smoking and body mass index alter the risk of multiple eye diseases and the effort to collect this information would be applicable to a number of complex traits affecting the eye [20]–[24]. Certainly, there are complex traits that would require collection of unique medical history information such as asking about atopic dermatitis in a study of retinal detachment; however, most of the answers would be negative and our impression is that the additional work is probably modest. Nonetheless, the documentation of other traits and biomorphic variables would modestly increase the cost of ascertainment, while also greatly increasing the value of the study.

One might argue that the use of clinic-based case-control studies should be minimized in favor of population-based case-control cohorts or family studies as more robust designs for avoiding possible confounding and biases [25], [26]. The major limitation of using these methods is the expense of identifying large numbers of subjects with and without the full spectrum of the primary trait. While population-based case-control studies may be ideal for gene discovery, such studies are rarely available because the full expense of the recruitment, diagnoses, and documentation must be covered by the research program. Family-based association is an excellent strategy for early-onset diseases, but is difficult to apply to age-related disease where the parents of the proband are often deceased [27]. Thus, clinic-based, case-control studies are a valuable tool for gene discovery because large numbers of subjects can be rapidly and efficiently ascertained and evaluated in a systematic manner. They are likely to continue to be a primary method used in genetic association studies of ocular traits.

The purpose of this study was to better understand the other complex ocular traits affecting the aging eye in the types of clinic-based, case-control studies currently being used by us and others to study the genetics of AMD and other traits. To this end, we documented the density of complex traits present in 100 consecutive patients age 60 or older attending a referral-based vitreoretinal clinic. This information was used to explore the additional information that might be gained by considering secondary ocular traits in genetic-epidemiology studies. The study revealed that most patients age 60 older have greater than 3 distinct diseases affecting their eyes, that most of these diseases were common ocular traits, and that they were typically present at a similar or higher frequency as population estimates. Controls without any other common and potentially hereditary eye diseases were rare. Rather than ignoring the potential confounding of secondary traits, we propose an alternative strategy in which subjects selected based on the presence of a disease be characterized for all other common diseases within that organ system. This approach would enable the detection of genetic risks for secondary traits while accounting for any stratification, confounding, or association between traits and other variables using standard statistical methods. The proposed “multiple-trait case-control study” employing several binary traits is analogous to traditional cohort studies in which the incidence of multiple quantitative traits are ascertained. These observations are important for the design of large-scale genome-wide association studies studying ocular traits and possibly for the study of other organ systems.

## Results

### Density of Complex Ocular Traits

In order to determine the density (number of traits per patient) of traits affecting the aging eye, the systematically documented diagnoses of 100 consecutive patients seen in the clinic for the first time for any reason from one retinal specialist were extracted from the medical record (Table 1). An average of 3.5 distinct diagnoses was observed in the 100 consecutive patients (Table 2). The 10 common complex traits listed in Table 1 accounted for 2.5 of these distinct diagnoses (Table 2). Only one of the 100 patients did not have at least 1 of the 10 common complex traits listed in Table 1. The density of complex traits in the 45 males was 2.5 compared to 2.6 in the 55 females (P = 0.70, Poisson regression).

**Table 1:** Common complex traits documented in 100 consecutive patients.

Ocular region or tissue	Disease name (abbreviation)	Evidence for hereditary component	Prevalence (%) in retina clinic patients	Estimate for Prevalence (%) in North America*
Cornea	Anterior basement membrane dystrophy (ABMD, OMIM 121820)	Minimal[57]	1	5
	Fuchs endothelial corneal dystrophy (Fuchs)	Strong[56], [58]	7	10
Lens	Cataract, age-related	Strong[59], [60]	96	50
	Pseudoexfoliation (PXE, OMIM 177650)	Strong[3]	3	5
Multiple	Glaucoma, open or closed angle or syndrome predisposing to glaucoma except for PXE	Strong[51], [61]	12	5
Vitreous	Posterior vitreous detachment (PVD, No OMIM number)	Unknown	54	50
Macula	Age-related macular degeneration (AMD)	Strong[1]	54	15
	Vitreomacular traction (Epiretinal membrane and macular hole, VMT; No OMIM number)	Minimal[62]–[64]	20	10
Retina	Occlusive retinal vascular disease (RVO; No OMIM number)	Minimal[65], [66]	7	2
	Retinal tears (with or without detachment; No OMIM number)	Strong[67]	5	3

* Please refer to the Discussion for an explanation of these approximate population estimates for individuals age 60 or older.

**Table 2:** Density of complex ocular traits in 100 consecutive patients age 60 or older arranged by presenting diagnosis.

Presenting Diagnosis	Number of Patients	Average number (standard error) of distinct diagnosis for the 20 observed traits	Average number (standard error) of the 10 complex traits in Table 1	Range of the 10 complex traits in Table 1
Age-related macular degeneration	47	3.7 (0.19)	2.9 (0.12)	1–5
Vitreomacular traction	22	3.7 (0.27)	2.8 (0.17)	2–4
Diabetic retinopathy	8	2.6 (0.18)	1.4 (0.18)	1–2
Retinal vascular occlusion (all BRVO)	4	4.8 (0.48)	3.8 (0.48)	3–5
Other	19	2.9 (0.30)	1.8 (0.22)	0–4
All patients	100	3.5 (0.13)	2.6 (0.10)	0–5

### Effect of Age on Density of Complex Ocular Traits

**Figure 1:**
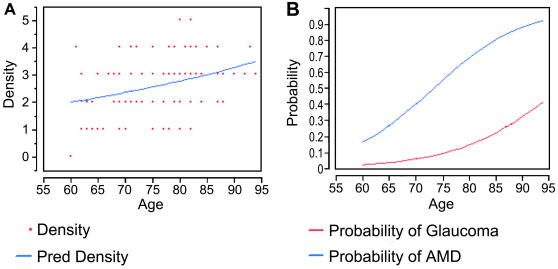
Scatter plot showing the density (number of distinct traits per patient) of the 10 complex ocular traits listed in Table 1 versus age for all 100 patients. A, The plotted points are the observed data and the line depicts the modeled relationship between age and density. B, The modeled probability of patients having AMD and glaucoma as a function of age using the logistic regression model.

A scatter plot of year of age versus the density of the ten complex ocular traits listed in Table 1 for each patient shows a trend toward an increase in density with age (Figure 1A). Poisson regression modeling showed an effect of age (P = 0.03) and estimated a rate of increase of 0.017±0.0074 log-number of complex traits per year across the age range represented in this study (ages 60–94). Using logistic regression, AMD (P<0.0001) and glaucoma (P = 0.01) were observed to increase with age (Figure 1B), while the frequency of posterior vitreous detachment (PVD) and vitreomacular traction (VMT) were not influenced by age. An effect of age was not observed for the combined frequency of the 8 complex traits remaining after removing AMD and glaucoma from the analysis (P = 0.66).

### Gender-Trait, and Trait-Trait Associations

Exploratory analyses were performed looking for gender-trait and trait-trait associations. Fisher's exact test was used to test for association with gender for all traits with at least 5 patients with the trait and 5 patients without the trait. No gender-trait association was observed for Fuchs, AMD, glaucoma, PVD, VMT, retinal vascular occlusion (RVO), or retinal tears. The low frequency of conditions thought to be associated with gender (e.g., VMT), may explain the failure to observe an association.

Trait-trait associations were assessed for each of the ten complex traits having at least 10 patients with and 10 patients without the trait (AMD, Glaucoma, PVD, VMT). The only significant association was AMD with VMT (P = 0.0009) in which VMT was protective. The distribution was as follows: AMD/VMT 4, AMD/No VMT 50, No AMD/VMT 16, and No AMD/No VMT 30. Since both eyes were always examined, it is unlikely that the VMT obscured the diagnosis of AMD, which usually affects both eyes. This observation probably reflects a referral bias in which patients with AMD and VMT are not referred for surgery for VMT, thus leading to a decrease in the number of subjects with AMD and VMT.

### Modeling of power to detect association with secondary traits in a genetic association study

We sought to determine the odds-ratio of a genetic effect that could be detected in a case-control study based on this clinic-based study. Using the frequencies of traits observed in the 100-person sample, we extrapolated to a similar hypothesized clinic-based sample of 2000 subjects. The 2000 subjects effectively form a cross-sectional study, where one defines diseased and not-diseased then analyzes with logistic regression models with estimation of odds-ratios for each genetic variant (a case-control study). The analysis enabled an estimation of the information on secondary traits that would be ignored in most ocular case-control studies. Specifically, we tested the hypothesis that comprehensive phenotyping of common ocular traits would enable discovery of genetic risks for secondary traits. The genotype data would be re-analyzed after re-coding cases and controls based on each secondary trait. The analysis showed that even this primitive ascertainment strategy, without any effort to increase the representation of less common traits, would have enabled detection of genetic risks for most of the traits (see Table 1 for observed frequencies of each trait). For example, at a significance level of 0.05, a modest genotypic effect of OR = 2.0 would have been detected in 6 of the 10 traits when the minor allele frequency was between 0.10 and 0.20 and 9 of the 10 traits when the minor allele frequency was 0.40 (Table 3). There was sufficient power to detect odds-ratios of 1.7, 1.7, 1.6, and 1.6 for the four most common traits glaucoma, PVD, AMD, and VMT, respectively. When the number of markers tested increases, as in the case of genome-wide association studies with a significance level of 0.00001, a modest genotypic effect of OR = 2.0 would have been detected in 4 out of the 10 traits when the minor allele frequency was 0.20 and 6 out of 10 traits when the minor allele frequency was 0.40 (Table 3).

**Table 3:** Odds-ratio for association between genotypes and traits that might be detected with a power of 0.8 at the specified minor allele frequency.

Complex ocular trait*	Number with trait	Number without trait	Odds-ratio** at a minor allele frequency of 0.1 ***			Odds-ratio at a minor allele frequency of 0.2***			Odds-ratio at a minor allele frequency of 0.4***		
			0.05	0.001	0.00001	0.05	0.001	0.00001	0.05	0.001	0.00001
ABMD	20	1980	3.25	5.20	7.90	2.70	4.10	6.10	2.50	4.00	6.30
Fuchs	140	1860	1.70	2.10	2.60	1.50	1.80	2.10	1.45	1.70	1.95
Cataract	1920	80	2.25	3.60	5.80	1.80	2.45	3.25	1.60	2.15	2.50
PXE	60	1940	2.10	2.80	3.60	1.80	2.35	2.90	1.70	2.15	2.70
Glaucoma	240	1760	1.55	1.80	2.10	1.40	1.60	1.80	1.35	1.50	1.70
PVD	1080	920	1.35	1.60	1.80	1.25	1.40	1.55	1.20	1.30	1.45
AMD	1080	920	1.35	1.55	1.70	1.25	1.40	1.50	1.20	1.30	1.40
VMT	400	1600	1.45	1.65	1.90	1.35	1.50	1.65	1.25	1.40	1.55
RVO	140	1860	1.70	2.05	2.45	1.50	1.80	2.10	1.45	1.70	1.95
Retinal tears	100	1900	1.80	2.30	2.80	1.60	2.00	2.35	1.50	1.85	2.15

* Abbreviations and definitions are given in Table 1.

** Power was estimated assuming a log-additive genetic model, significance levels of 0.05, 0.001, and 0.00001, complete linkage disequilibrium between the single nucleotide polymorphism (SNP) studied and the genetic variant underlying the association, and the population prevalence estimates shown in Table 1.

*** The three significance levels, namely 0.05, 0.001, and 0.00001, refer to testing one variant, multiple variants in a single gene, and variants across the genome, respectively.

## Discussion

We show that the average patient age 60 or older has at least 3.5 distinct ocular diseases or traits, and that the majority of these diseases arise from common complex traits affecting the eye. Further, we show that some traits steadily increase with age, but that others do not appear to increase after the age of 60 years. Finally, we show that comprehensive documentation of secondary common complex traits affecting the aging eye would substantially increase the value of a genome-wide genetic association study.

We chose to use clinic-based cases and controls from a subspecialty clinic, because this represents the most common method used to ascertain patients for genetic case-control studies of eye diseases. Such samples are easily collected during the course of routine patient care. Further, large numbers of affected subjects with broad representation of the spectrum of disease and its complications can be collected in this manner, which is typically not possible with population-based approaches due to financial constraints. It is important to realize the limitations of clinic-based, case-control studies. The frequencies of traits do not necessarily reflect the underlying population prevalence and they tend to over-estimate population parameters such as relative risks and attributable fractions. Further, replication is essential for all genetic association results, regardless of the study design [28].

Additional concerns in the use of case-control studies are the potential for selection bias and the possibility of confounding due to unmeasured differences between case and control status that are associated with genetic exposures. One manner is which this could arise might be from referral patterns to the specific physicians from which the case or control subjects are being collected. Thus, an additional benefit of documentation of secondary complex traits affecting the eye would be to account for such effects using standard statistical techniques. At this time it is largely unknown if this type of confounding exists in genetic association studies of ophthalmic or other traits. Thus, our identification of association between vitreomacular traction and AMD is of particular interest. The observation that patients with vitreomacular traction were less likely to have AMD, was unexpected because previous studies have suggested it might be a risk factor for exudative AMD [11], [13], [29]. Our observation likely is due to referral biases, where patients with vitreomacular traction who also had AMD were not referred for surgical management.

Even though the examiner of the 100 consecutive subjects in this study routinely documents all ocular traits including the ten in Table 1, we recognize that traits are missed due to the distractions that occur in a busy clinic. Thus, our estimate of the density of common complex traits is very likely under-estimated. Prospective examination with photographic documentation and quantitative grading would be preferred when possible for genetic association studies. Nonetheless, we sought to compare the observed frequency of complex traits in our 100 patients to the previous reports of prevalence of the ten common complex traits (Table 1), because this information is important in designing future studies using case-control studies.

Prevalence studies of corneal guttae have not been performed in North American populations. The Reykjavik study estimated an overall 9% prevalence in a Caucasian population, while a study on Asians estimated 3.5% to 6.5% depending on the population [30], [31]. ABMD may be present in about 5% of the population, based on a prior prevalence study in North Americans [32]. The prevalence of cataract was estimated to be about 50% [33], [34]. The much higher prevalence in our patients is due to the difference in definition of the age-related lens changes to diagnose a cataract, since almost all patients have some age-related lens change by the age of 60. Use of one of the quantitative scales would be preferred for a genetic association study [35]. The prevalence of pseudoexfoliation varies greatly across populations, and no previous studies appeared to be a good fit the population of Minnesota and an estimate of 5% was assumed based on other populations [36]–[38].

The much higher prevalence of AMD in our patients, compared to the population estimate of 15%, is due to referral bias, since this was the leading cause of presenting diagnosis [39]. The population prevalence of glaucoma is about 5% based on the Beaver Dam study [40]. The observed prevalence of posterior vitreous separation in our study was similar to published reports [41]. The prevalence of epiretinal membranes and retinal vascular occlusion in the Blue Mountains Eye Study was about 10%, and 2% respectively [42], [43]. The prevalence of retinal tears in autopsy eyes was 3.3% [44]. These latter three traits are also common reasons for referral. Thus, with the exception of the traits representing common reasons for referral to the examiner and the lower threshold for diagnosis of cataract, the observed prevalence of these 10 complex traits is a reasonable approximation to published reports (Table 1). It was notable how little prevalence information, population-based or otherwise, was actually available for most common ocular traits in North America.

Genetic association studies on age-related ocular traits often use age 60 or older for controls, under the assumption that subjects in this age range are unlikely to develop the trait under study at a later age. Our observation of a steady increase in the density of complex traits with age might be explained by only older subjects symptomatic or having vision threatening eye diseases coming to the clinic. We have no method for controlling for this possibility, but our observations are consistent with the already established increase in disease prevalence for AMD, glaucoma, and RVO [40], [43], [45]. Further, for PVD which is known to stabilize with age, we did not observe an age-dependent increase [46]. Thus, the selection of an age-cut off for controls should be selected based upon the age-dependent prevalence of the disease or preferably either frequency or exact matched for age. The commonly held opinion that age 60 is the lowest acceptable minimal age for controls used in studies on AMD, glaucoma, and other eye traits is not supported by our data.

In addition to these complex traits affecting the eye, there are a number of quantitative biomorphic and biochemical variables of interest in eye development and disease risk. For example, central corneal thickness has been associated with risk of development of glaucoma and many investigators believe that macular pigment and macular autofluorescence alter the risk of macular degeneration [17], [19], [47]. Although there are few studies exploring the heritability of such quantitative variables in humans, available evidence suggests that biomorphic ocular variables are highly hereditary [48]–[52]. In addition to demographic and epidemiological variables such as dietary and drug exposures, these variables should be considered for inclusion in a comprehensive study of complex ocular traits.

The density of complex traits in these patients and our modeling studies suggest that future studies of complex ocular traits would benefit from the systematic documentation of secondary traits. An ascertainment strategy that focused on the diseases of highest priority (e.g., AMD, glaucoma, Fuchs, cataract) could be employed to optimize the case-control study for detection of other traits of secondary interest. There is substantial evidence that many of the common traits have genetic influences, as illustrated in Table 1. Of course, the lack of strong evidence does not imply the absence of a genetic effect as was recently demonstrated for pseudoexfoliation [3]. Inclusion of biomorphic and biochemical endpoints would further expand the value of the study. This type of approach would offer advantages over the standard approach looking at a single trait including the ability to look for shared genetic risks directly, the ability to detect trait-trait association, and overall lower cost. Although our model for this study was the aging eye, there is evidence that similar concepts apply to other organs such as the skin [53].

A number of study designs are available to study common complex traits. The Wellcome Trust Case Control Consortium used a pool of unexamined subjects as the controls for each of the seven traits studied [54]. While this is a reasonable approach, it does not appear to have any major advantages over sequentially studying secondary traits in a case-control study. For example, both could be influenced by referral biases and geographic differences in genetics or exposures. Further, the unexamined control strategy results in loss of power for very common traits, because a certain proportion of the controls have disease. The approach we are suggesting can control for the various biases in subject ascertainment and exposures, maximizes power because all subjects are phenotyped, and minimizes genotyping costs because most (or all) subjects have one or more diseases of clinical interest.

## Methods

### Patient chart review

This retrospective chart review was approved by the institutional review board of the Mayo Clinic and patient consent was not required. The data was extracted twice and any discrepancies resolved by the first author. The initial visit was reviewed first and subsequent visits reviewed to look for diseases present at the first examination, but that were not documented. Although this study is retrospective, the retina specialist performed a systematic and comprehensive eye examination documenting all ocular diagnoses, including the complex traits listed in Table 1 on all patients examined during their initial visit. Note that these common traits extracted from the medical record were selected in advance, prior to the start of the study, because they are documented on most visits due to their relevance to the performance or complications of surgery and/or potential impact on vision. Thus, the number of diagnoses overlooked is expected to be modest. Age and gender were recorded in order to stratify the density of complex traits by these two variables. The final diagnosis underlying the reason for the patient's initial consultation was recorded as the presenting diagnosis. Ethnicity was not considered in the analysis because over 90% of the patients were Caucasian, reflecting the frequency of this population in southeastern Minnesota.

The diagnostic criteria followed standard clinical criteria. The diagnosis of AMD was made based on a published classification system [55]. Fuchs was diagnosed based on having two or more central guttae similar to a previously published scale [56]. Cataract was diagnosed based on any discoloration or opacification of the nucleus or cortex or prior cataract surgery, PVD based on the presence of a Weiss ring or a visible posterior hyaloid by examination or ultrasound. Glaucoma was based on optic nerve damage attributed to glaucoma. The other diseases were documented using standard clinical criteria.

Primary complex traits and all distinct diagnoses were recorded. Complications of primary diseases, such as retinal edema from branch retinal vein occlusions, were not extracted. Refractive errors were not recorded because of the absence of refractive status prior to cataract surgery and the absence of important refractive components such as axial length. Posterior staphylomata from high myopia were not recorded because of the lack of an ophthalmic ultrasound to record the diagnosis accurately, but myopic degeneration of the RPE and retina was recorded as an accurate marker of myopic degeneration. Secondary diagnoses such as epiretinal membranes from trauma or glaucoma from surgical complications were not included. Drusen insufficient to meet the diagnosis of AMD were not considered a diagnosis. Extraocular conditions such as blepharitis and ptosis were not extracted.

### Statistical analysis

This study was primarily descriptive with the goal of determining the density (number per patient) of complex ocular traits affecting a clinic-based study of patients age 60 or older. Poisson regression models were fit to assess the effect of age and gender on the density of the 10 complex ocular traits in Table 1 and likelihood ratio rests were used to assess the significance of age and gender. Fisher's exact tests were used to test for association between the presence or absence of a trait and gender (trait-gender) and another trait (trait-trait). Logistic regression was used to model the association between traits (presence or absence) and age and tests for association were completed using likelihood ratio tests. Significance results (p-values) are presented without correction for multiple testing.

### Modeling of power to detect association with secondary traits in a genetic association study

The traits observed in the 100 consecutive patients were extrapolated to create a hypothetical clinic-based study of 2000 patients. The odds-ratio that could be detected with power of 0.8 given the frequency of each trait and a minor allele frequency of 0.1, 0.2, and 0.4 were calculated. Power for the main analysis of AMD and the secondary eye traits was computed assuming a log-additive genetic model, complete linkage disequilibrium between the SNP and the causative variant, and a population-based estimate of the prevalence of each trait. Varying significance levels were used to adjust for multiple testing of many variants, namely, 0.05 for one variant, 0.001 for multiple variants in a single gene, and 0.00001 for multiple variants across the genome.

## References

[1] Edwards AO, Malek G (2007). Molecular genetics of AMD and current animal models.. Angiogenesis in press.

[2] Matsuda A, Ebihara N, Kumagai N, Fukuda K, Ebe K (2007). Genetic polymorphisms in the promoter of the interferon gamma receptor 1 gene are associated with atopic cataracts.. Invest Ophthalmol Vis Sci.

[3] Thorleifsson G, Magnusson KP, Sulem P, Walters GB, Gudbjartsson DF (2007). Common sequence variants in the LOXL1 gene confer susceptibility to exfoliation glaucoma.. Science.

[4] Freedman ML, Haiman CA, Patterson N, McDonald GJ, Tandon A (2006). Admixture mapping identifies 8q24 as a prostate cancer risk locus in African-American men.. Proc Natl Acad Sci U S A.

[5] McPherson R, Pertsemlidis A, Kavaslar N, Stewart A, Roberts R (2007). A common allele on chromosome 9 associated with coronary heart disease.. Science.

[6] Edwards AO, Ritter R, Abel KJ, Manning A, Panhuysen C (2005). Complement factor H polymorphism and age-related macular degeneration.. Science.

[7] Li M, Atmaca-Sonmez P, Othman M, Branham KE, Khanna R (2006). CFH haplotypes without the Y402H coding variant show strong association with susceptibility to age-related macular degeneration.. Nat Genet.

[8] Conley YP, Jakobsdottir J, Mah T, Weeks DE, Klein R (2006). CFH, ELOVL4, PLEKHA1 and LOC387715 genes and susceptibility to age-related maculopathy: AREDS and CHS cohorts and meta-analyses.. Hum Mol Genet.

[9] Maller J, George S, Purcell S, Fagerness J, Altshuler D (2006). Common variation in three genes, including a noncoding variant in CFH, strongly influences risk of age-related macular degeneration.. Nat Genet.

[10] Haines JL, Hauser MA, Schmidt S, Scott WK, Olson LM (2005). Complement factor H variant increases the risk of age-related macular degeneration.. Science.

[11] Krebs I, Brannath W, Glittenberg C, Zeiler F, Sebag J (2007). Posterior vitreomacular adhesion: a potential risk factor for exudative age-related macular degeneration?. Am J Ophthalmol.

[12] Schmidt JC, Mennel S, Horle S, Meyer CH (2006). High incidence of vitreomacular traction in recurrent choroidal neovascularisation after repeated photodynamic therapy.. Br J Ophthalmol.

[13] Ondes F, Yilmaz G, Acar MA, Unlu N, Kocaoglan H (2000). Role of the vitreous in age-related macular degeneration.. Jpn J Ophthalmol.

[14] Rao GP, Kaye SB, Agius-Fernandez A (1998). Central corneal endothelial guttae and age-related macular degeneration: is there an association?. Indian J Ophthalmol.

[15] Lee MM, Ritter R, Hirose T, Vu CD, Edwards AO (2003). Snowflake vitreoretinal degeneration: follow-up of the original family.. Ophthalmology.

[16] Hejtmancik JF, Jiao X, Li A, Sergeev YV, Ding X (2008). Mutations in KCNJ13 cause autosomal dominant snowflake vitreoretinal degeneration.. Am J Hum Genet in press.

[17] Brandt JD, Beiser JA, Kass MA, Gordon MO (2001). Central corneal thickness in the Ocular Hypertension Treatment Study (OHTS).. Ophthalmology.

[18] Szijarto Z, Schvoller M, Poto L, Kuhn F, Kovacs B (2007). Pseudophakic retinal detachment after phacoemulsification.. Ann Ophthalmol (Skokie).

[19] Schmitz-Valckenberg S, Bindewald-Wittich A, Dolar-Szczasny J, Dreyhaupt J, Wolf S (2006). Correlation between the area of increased autofluorescence surrounding geographic atrophy and disease progression in patients with AMD.. Invest Ophthalmol Vis Sci.

[20] Hyman LG, Lilienfeld AM, Ferris FLd, Fine SL (1983). Senile macular degeneration: a case-control study.. Am J Epidemiol.

[21] Zoega GM, Fujisawa A, Sasaki H, Kubota A, Sasaki K (2006). Prevalence and risk factors for cornea guttata in the Reykjavik Eye Study.. Ophthalmology.

[22] Clemons TE, Milton RC, Klein R, Seddon JM, Ferris FL (2005). Risk factors for the incidence of Advanced Age-Related Macular Degeneration in the Age-Related Eye Disease Study (AREDS) AREDS report no. 19.. Ophthalmology.

[23] Nakano T, Tatemichi M, Miura Y, Sugita M, Kitahara K (2005). Long-term physiologic changes of intraocular pressure: a 10-year longitudinal analysis in young and middle-aged Japanese men.. Ophthalmology.

[24] Budenz DL, Anderson DR, Feuer WJ, Beiser JA, Schiffman J (2006). Detection and prognostic significance of optic disc hemorrhages during the Ocular Hypertension Treatment Study.. Ophthalmology.

[25] Hopper JL, Bishop DT, Easton DF (2005). Population-based family studies in genetic epidemiology.. Lancet.

[26] Clayton D, McKeigue PM (2001). Epidemiological methods for studying genes and environmental factors in complex diseases.. Lancet.

[27] Laird NM, Lange C (2006). Family-based designs in the age of large-scale gene-association studies.. Nat Rev Genet.

[28] Dahlman I, Eaves IA, Kosoy R, Morrison VA, Heward J (2002). Parameters for reliable results in genetic association studies in common disease.. Nat Genet.

[29] Weber-Krause B, Eckardt U (1996). [Incidence of posterior vitreous detachment in eyes with and without age-related macular degeneration. An ultrasonic study].. Ophthalmologe.

[30] Khan AO (2006). Reykjavik Eye Study and cornea guttata.. Ophthalmology.

[31] Kitagawa K, Kojima M, Sasaki H, Shui YB, Chew SJ (2002). Prevalence of primary cornea guttata and morphology of corneal endothelium in aging Japanese and Singaporean subjects.. Ophthalmic Res.

[32] Werblin TP, Hirst LW, Stark WJ, Maumenee IH (1981). Prevalence of map-dot-fingerprint changes in the cornea.. Br J Ophthalmol.

[33] Mitchell P, Cumming RG, Attebo K, Panchapakesan J (1997). Prevalence of cataract in Australia: the Blue Mountains eye study.. Ophthalmology.

[34] Klein BE, Klein R, Linton KL (1992). Prevalence of age-related lens opacities in a population. The Beaver Dam Eye Study.. Ophthalmology.

[35] Chylack LT, Wolfe JK, Singer DM, Leske MC, Bullimore MA (1993). The Lens Opacities Classification System III. The Longitudinal Study of Cataract Study Group.. Arch Ophthalmol.

[36] Young AL, Tang WW, Lam DS (2004). The prevalence of pseudoexfoliation syndrome in Chinese people.. Br J Ophthalmol.

[37] Arnarsson A, Damji KF, Sverrisson T, Sasaki H, Jonasson F (2007). Pseudoexfoliation in the Reykjavik Eye Study: prevalence and related ophthalmological variables.. Acta Ophthalmol Scand.

[38] Mitchell P, Wang JJ, Hourihan F (1999). The relationship between glaucoma and pseudoexfoliation: the Blue Mountains Eye Study.. Arch Ophthalmol.

[39] Friedman DS, O'Colmain BJ, Munoz B, Tomany SC, McCarty C (2004). Prevalence of age-related macular degeneration in the United States.. Arch Ophthalmol.

[40] Klein BE, Klein R, Sponsel WE, Franke T, Cantor LB (1992). Prevalence of glaucoma. The Beaver Dam Eye Study.. Ophthalmology.

[41] Snead MP, Snead DR, Richards AJ, Harrison JB, Poulson AV (2002). Clinical, histological and ultrastructural studies of the posterior hyaloid membrane.. Eye.

[42] Mitchell P, Smith W, Chey T, Wang JJ, Chang A (1997). Prevalence and associations of epiretinal membranes. The Blue Mountains Eye Study, Australia.. Ophthalmology.

[43] Mitchell P, Smith W, Chang A (1996). Prevalence and associations of retinal vein occlusion in Australia. The Blue Mountains Eye Study.. Arch Ophthalmol.

[44] Straatsma BR (1980). Peripheral retinal tears: classification, prevalence and principles of management.. Aust J Ophthalmol.

[45] Klein R, Klein BE, Linton KL (1992). Prevalence of age-related maculopathy. The Beaver Dam Eye Study.. Ophthalmology.

[46] Snead MP, Snead DR, Mahmood AS, Scott JD (1994). Vitreous detachment and the posterior hyaloid membrane: a clinicopathological study.. Eye.

[47] Whitehead AJ, Mares JA, Danis RP (2006). Macular pigment: a review of current knowledge.. Arch Ophthalmol.

[48] He M, Wang D, Zheng Y, Zhang J, Yin Q (2008). Heritability of anterior chamber depth as an intermediate phenotype of angle-closure in Chinese: the Guangzhou Twin Eye Study.. Invest Ophthalmol Vis Sci.

[49] Zhu G, Hewitt AW, Ruddle JB, Kearns LS, Brown SA (2007). Genetic Dissection of Myopia Evidence for Linkage of Ocular Axial Length to Chromosome 5q.. Ophthalmology.

[50] Peet JA, Cotch MF, Wojciechowski R, Bailey-Wilson JE, Stambolian D (2007). Heritability and familial aggregation of refractive error in the Old Order Amish.. Invest Ophthalmol Vis Sci.

[51] van Koolwijk LM, Despriet DD, van Duijn CM, Pardo Cortes LM, Vingerling JR (2007). Genetic contributions to glaucoma: heritability of intraocular pressure, retinal nerve fiber layer thickness, and optic disc morphology.. Invest Ophthalmol Vis Sci.

[52] Chen CY, Scurrah KJ, Stankovich J, Garoufalis P, Dirani M (2007). Heritability and shared environment estimates for myopia and associated ocular biometric traits: the Genes in Myopia (GEM) family study.. Hum Genet.

[53] Sandilands A, O'Regan GM, Liao H, Zhao Y, Terron-Kwiatkowski A (2006). Prevalent and rare mutations in the gene encoding filaggrin cause ichthyosis vulgaris and predispose individuals to atopic dermatitis.. J Invest Dermatol.

[54] Zeggini E, Weedon MN, Lindgren CM, Frayling TM, Elliott KS (2007). Replication of genome-wide association signals in UK samples reveals risk loci for type 2 diabetes.. Science.

[55] Klein R, Davis MD, Magli YL, Segal P, Klein BE (1991). The Wisconsin age-related maculopathy grading system.. Ophthalmology.

[56] Krachmer JH, Purcell JJ, Young CW, Bucher KD (1978). Corneal endothelial dystrophy. A study of 64 families.. Arch Ophthalmol.

[57] Boutboul S, Black GC, Moore JE, Sinton J, Menasche M (2006). A subset of patients with epithelial basement membrane corneal dystrophy have mutations in TGFBI/BIGH3.. Hum Mutat.

[58] Sundin OH, Broman KW, Chang HH, Vito EC, Stark WJ (2006). A common locus for late-onset Fuchs corneal dystrophy maps to 18q21.2-q21.32.. Invest Ophthalmol Vis Sci.

[59] Hejtmancik JF, Kantorow M (2004). Molecular genetics of age-related cataract.. Exp Eye Res.

[60] Klein AP, Duggal P, Lee KE, O'Neill JA, Klein R (2005). Polygenic effects and cigarette smoking account for a portion of the familial aggregation of nuclear sclerosis.. Am J Epidemiol.

[61] Nemesure B, Jiao X, He Q, Leske MC, Wu SY (2003). A genome-wide scan for primary open-angle glaucoma (POAG): the Barbados Family Study of Open-Angle Glaucoma.. Hum Genet.

[62] Lalin SC, Chang S, Flynn H, Von Fricken M, Del Priore LV (2004). Familial idiopathic macular hole.. Am J Ophthalmol.

[63] Scott A, Strouthidis NG, Robson AG, Forsyth J, Maher ER (2007). Bilateral epiretinal membranes in Gorlin syndrome associated with a novel PTCH mutation.. Am J Ophthalmol.

[64] Black GC, Mazerolle CJ, Wang Y, Campsall KD, Petrin D (2003). Abnormalities of the vitreoretinal interface caused by dysregulated Hedgehog signaling during retinal development.. Hum Mol Genet.

[65] Girmens JF, Scheer S, Heron E, Sahel JA, Tournier-Lasserve E (2008). Familial central retinal vein occlusion.. Eye.

[66] Weger M, Renner W, Steinbrugger I, Cichocki L, Temmel W (2005). Role of thrombophilic gene polymorphisms in branch retinal vein occlusion.. Ophthalmology.

[67] Go SL, Hoyng CB, Klaver CC (2005). Genetic risk of rhegmatogenous retinal detachment: a familial aggregation study.. Arch Ophthalmol.

